# EZB-Schuldverschreibungen — neue Verwendung für ein altes Instrument?

**DOI:** 10.1007/s10273-021-3008-0

**Published:** 2021-09-21

**Authors:** Arne Hansen, Dirk Meyer

**Affiliations:** Institut für Volkswirtschaftslehre, Helmut-Schmidt-Universität, Holstenhofweg 85, 22043 Hamburg, Deutschland

**Keywords:** E44, E52, E58, E63, H63

## Abstract

Als Reaktion auf die Corona-Krise haben die Notenbanken des Eurosystems ihre Anleihekäufe im Rahmen des APP-Programms weiter aufgestockt und um das PEPP-Programm erweitert. Diese im Zusammenspiel mit weiteren Maßnahmen der „außergewöhnlichen Geldpolitik“ geschaffene zusätzliche Liquidität müsste bei anhaltend höherem Inflationsdruck zurückgeführt werden. Als eine Neutralisierung auf dem Entstehungsweg wäre ein umfänglicher Verkauf von Anleihebeständen naheliegend. Mit den resultierenden Kursverlusten gingen allerdings abschreibungsbedingte Instabilitäten im Finanzsektor und insbesondere für Krisenstaaten problematische Zinsanstiege bei Staatsanleihen einher. Der Beitrag analysiert als alternative Möglichkeit die Emission von Schuldverschreibungen durch die EZB zur Liquiditätsabschöpfung.

## Abstract (en)

In response to the coronavirus crisis, the central banks of the Eurosystem have further increased their APP bond purchases and supplemented them with purchases under the PEPP programme. Together with other unconventional monetary policy measures, this generated additional liquidity would have to be reduced in the case of sustained higher inflationary pressure. The straightforward solution for the ECB would be to sell the once purchased bonds, as they are the source of the extra liquidity. However, the associated writeoffs would result in financial sector instabilities and interest rate increases on government bonds, which would be particularly problematic for crisis-hit countries. This contribution analyses the issuance of debt certificates by the ECB as an alternative way of absorbing liquidity.

Seit Jahresbeginn ist das Thema Preissteigerung zurück, wobei die langfristigen Inflationsgefahren schwer prognostizierbar sind. Fakt ist eine Überschussliquidität in Höhe von rund 3,5 Billionen Euro, die die Banken irgendwann für die Kreditvergabe verwenden mögen, also dem Wirtschaftskreislauf zuführen können. Deshalb scheint der Blick auf eine mögliche und etwaig nötige Rückholung des überschüssigen Geldes durch die EZB geboten. Eine Veräußerung der im Rahmen der außergewöhnlichen Geldpolitik angekauften Anleihen wäre eine naheliegende Möglichkeit. Angesichts des großen Umfangs der erworbenen Wertpapiere – davon mehr als 3 Billionen Euro an Staatsanleihen – wären jedoch erhebliche Kursverluste und entsprechende Zinsanstiege wahrscheinlich. Steigende Zinslasten, ein den Krisenstaaten versperrter Kreditzugang und ein Wertberichtigungsbedarf bei Banken und Versicherungen könnten eine erneute Staaten- und Bankenkrise hervorrufen. Alternative bzw. flankierende Handlungsoptionen wie ein Anheben der Leitzinsen, eine Erhöhung der Mindestreservesätze oder, unkonventioneller, ein Schuldenerlass für die teilweise hochverschuldeten Mitgliedstaaten mittels einer Streichung von Staatsschulden im Portfolio des Eurosystems sind nicht ohne gravierende Nebenwirkungen (Hansen und Meyer, 2020a, 2021a). Stattdessen könnten die Euro-Mitgliedstaaten im Verhältnis ihres EZB-Kapitalanteils Staatsschuldtitel am Kapitalmarkt begeben, um die Erlöse auf einem Konto der EZB – gegebenenfalls temporär – stillzulegen (Bini Smaghi, 2009, 12). Jedoch steht mit dieser Maßnahme die Unabhängigkeit der Notenbank infrage. Um einerseits nachteilige Wirkungen der obigen Instrumente bzw. Maßnahmen zu vermeiden und andererseits im Rahmen der Legalität zu handeln, bestände für die EZB alternativ die Möglichkeit, eigene Schuldpapiere zu emittieren.[1]

**Dr. Arne Hansen** ist wissenschaftlicher Mitarbeiter an der Helmut-Schmidt-Universität Hamburg.

**Prof. Dr. Dirk Meyer** ist Inhaber des Lehrstuhls für Ordnungsökonomik an der Helmut-Schmidt-Universität, Universität der Bundeswehr Hamburg.

## Was sind Notenbank-Schuldverschreibungen?

Eine Notenbank-Schuldverschreibung ist ein von der Zentralbank emittiertes Wertpapier, das auf dem Kapitalmarkt gegen Zentralbankgeld (M0) eingewechselt wird. Demnach zählt es nicht zur Geldbasis, die ja gerade reduziert werden soll.[2] Banken entrichten den Kaufpreis in

1 So auch De Grauwe (2021): „In that case if the inflation surges beyond 2 %, it will have to reduce the amount of outstanding money base by either selling government bonds or issuing its own interest bearing bonds..."

2 Allerdings stellen „Geldmarktpapiere ... eine Verbriefung von Zentralbankgeld dar, wenn die Bundesbank ... verpflichtet ist, alle Verbindlichkeiten aus Papieren zu erfüllen, die auf ihre eigene Initiative hin emittiert werden" (Deutsche Bundesbank, 1997, 48).

Zentralbankgeld, was ihre Überschussliquidität mindert. Folglich handelt es sich vereinfacht um einen Passivtausch in der Bilanz der Notenbank. Damit ersetzt die Notenbank Zentralbankgeld gegen eine weniger liquide Schuldverschreibung, die im Regelfall eine feste Laufzeit hat und mit einem Zinscoupon ausgestattet ist. Je nach Fälligkeit kann diese Wertschrift auch als geldnahes Substitut verwendet werden. Denkbar sind Laufzeiten von wenigen Tagen bis hin zu einem unendlichen Wertpapier ohne Tilgung.[3] Kurzläufer dienen demnach eher der geldpolitischen Feinsteuerung. Aufgrund des Zinscoupons führt die Emission einer Notenbank-Schuldverschreibung im Vergleich zu einer Erhöhung des Leitzinses oder der Mindestreserveanforderungen zu einer Belastung der Notenbank, senkt also die Seigniorage.

Mit der Emission von Notenbank-Schuldverschreibungen können verschiedene Ziele verfolgt werden (Rule, 2011; Gray und Pongsaparn, 2015; Hardy, 2020; Boonstra, 2019):

- Erstens rechnen sie zu den strukturellen geldpolitischen Operationen, mit denen der Bedarf der Geschäftsbanken an Zentralbankgeld langfristig beeinflusst werden kann (Deutsche Bundesbank, 2019, 177 f.). Speziell zur Abschöpfung einer Überschussliquidität lassen sie sich einsetzen. Die Hintergründe einer Überschussliquidität sind vielfältig. Mit dem Einsatz von Kurzläufern kann die Notenbank eine geldpolitische Feinsteuerung vornehmen. Darüber hinaus lässt sich die Absorption eines ausländischen Kapitalzuflusses (Devisen) vornehmen bzw. der Aufbau von Währungsreserven finanzieren. Schließlich dienen sie im Rahmen einer unkonventionellen Form der Ausweitung der Geldbasis (QE) deren Rückführung, um unerwünschte Kapitalmarktreaktionen (Kursverluste/Renditenanstieg bei Staatsanleihen) zu vermeiden. Indem Notenbank-Schuldverschreibungen unbegrenzt verfügbar und handelbar sind, zudem nicht notwendig an den Bankensektor knüpfen und eine breite Transformation gewährleisten, ist ein effektiver Einsatz dieses Instruments für geldpolitische Zwecke gesichert. Darüber hinaus gewährleistet dieses Instrument der Zentralbank ein hohes Maß an Autonomie (Rule, 2011, 13 f.; Gray und Pongsaparn, 2015, 5; Buzeneca und Maino, 2007, 27).

- Zweitens dürften Notenbank-Schuldverschreibungen infolge der Ausfallsicherheit zur neuen risikolosen Benchmark werden. Gerade in einer Währungsunion, die wie die Eurozone durch relativ heterogene Mitgliedstaaten geprägt ist, kommt dem Aufbau einer gemeinschaftsweiten Zinsstrukturkurve durch die Schaffung eines risikolosen Vermögenswerts eine besondere Bedeutung zu. Sie ist wichtig, um eine reibungslose Transmission einer gemeinschaftsweiten Geldpolitik sicherzustellen. Ihr derzeitiges Fehlen ist mit ein Indiz dafür, dass die Kapitalmärkte national fragmentiert sind und eine Kapitalmarktunion noch nicht geschaffen wurde (Boonstra, 2019, 4 f.; Tonveronachi, 2014, 4 und 6; ders., 2018, 10 f.). EZB-Schuldverschreibungen (EZB-Bonds) mit einer Duration über das gesamte Fälligkeitsspektrum könnten diesen Mangel beheben.[4]

- Drittens dient die Emission der Bereitstellung sicherer Anleihen. Da Notenbank-Schuldverschreibungen überaus sicher sind, sicherer als eine vom Staat emittierte Anleihe, dürften insbesondere Banken und Versicherungen zu den Nachfragenden zählen. Dieser Faktor könnte zudem eine Haltung als Reservewährung befördern, weshalb eine Notenbank wie die EZB ein Interesse an der Emission von Notenbank-Schuldverschreibungen haben könnte (Boonstra, 2019, 5 f.).

## Fallstudie: stilisierte EZB-Bilanzen

Abhängig von der geldpolitischen Zielsetzung und den Rahmenbedingungen können Notenbank-Schuldverschreibungen in unterschiedlicher Weise als Offenmarktoperation eingesetzt werden. Die folgenden Szenarien lassen sich am Beispiel der EZB-Bilanz illustrieren. Siehe hierzu Abbildung 1, in der die von einer EZB-Bonds-Emission potenziell betroffenen Positionen hervorgehoben wurden.

### Passivtausch zur Abschöpfung von Überschussliquidität der Geschäftsbanken

Die Notenbank emittiert eigene Schuldverschreibungen. Indem die Geschäftsbanken diese am Kapitalmarkt ankaufen, mindert sich ihre Überschussliquidität. Das Ergebnis ist ein Passivtausch in der Bilanz der Notenbank. Je nach Laufzeit und Prolongation der Notenbank-Schuldverschreibungen wird Liquidität eingefroren. Für die EZB gelte hierbei der besondere Umstand, dass ein Verkauf der im Rahmen der QE-Programme angekauften Staatsanleihen aufgrund von drohenden Instabilitäten kaum infrage kommt und deshalb EZB-Bonds emittiert werden.

3 Denkbar wäre sogar eine Übernacht-Schuldverschreibung, die den fließenden Übergang zur Einlagefazilität deutlich werden lässt. Der Wert eines Wertpapiers mit unendlicher Laufzeit ergibt sich aus dem Kurswert = Zinscoupon / Zinssatz. Da sich die Marktrendite laufend ändert, kann der Wert im Zeitablauf erheblich steigen oder sinken.

4 Hardy (2020, 25) bewertet hingegen nur den kurzfristigen Teil der Zinsstrukturkurve als noch nicht mit einer gemeinschaftsweiten risikolosen Benchmark abgedeckt.

Abbildung 1

**EZB-Schuldverschreibungen in der konsolidierten Bilanz des Eurosystems**

| Aktiva | Passiva | |
|---|---|---|
| 1 Gold und Goldforderungen | 1 Banknotenumlauf | |
| 2 Forderungen in Fremdwährung an Ansässige außerhalb des Euro-Währungsgebiets | 2 Verbindlichkeiten in Euro aus geldpolitischen Operationen gegenüber Kreditinstituten im Euro-Währungsgebiet | betr. Position sinkt |
| 3 Forderungen in Fremdwährung an Ansässige im Euro-Währungsgebiet | 2.1 Einlagen auf Girokonten (einschließlich Mindestreserveguthaben) | |
| | 2.2 Einlagefazilität | |
| 4 Forderungen in Euro an Ansässige außerhalb des Euro-Währungsgebiets | 2.3 Termineinlagen | |
| | 2.4 Feinsteuerungsoperationen in Form von befristeten Transaktionen | |
| 5 Forderungen in Euro aus geldpolitischen Operationen an Kreditinstitute im Euro-Währungsgebiet | 2.5 Verbindlichkeiten aus Margenausgleich | |
| 5.1 Hauptrefinanzierungsgeschäfte | | |
| 5.2 Längerfristige Refinanzierungsgeschäfte | 3 Sonstige Verbindlichkeiten in Euro gegenüber Kreditinstituten im Euro-Währungsgebiet | |
| 5.3 Feinsteuerungsoperationen in Form von befristeten Transaktionen | | |
| 5.4 Strukturelle Operationen in Form von befristeten Transaktionen | 4 Verbindlichkeiten aus der Begebung von Schuldverschreibungen | Position steigt |
| 5.5 Spitzenrefinanzierungsfazilität | 5 Verbindlichkeiten in Euro gegenüber sonstigen Ansässigen im Euro-Währungsgebiet | |
| 5.6 Forderungen aus Margenausgleich | 5.1 Einlagen von öffentlichen Haushalten | |
| 6 Sonstige Forderungen in Euro an Kreditinstitute im Euro-Währungsgebiet | 5.2 Sonstige Passiva | |
| 7 Wertpapiere in Euro von Ansässigen im Euro-Währungsgebiet | 6 Verbindlichkeiten in Euro gegenüber Ansässigen außerhalb des Euro-Währungsgebiets | |
| 7.1 Zu geldpolitischen Zwecken gehaltene Wertpapiere | | |
| 7.2 Sonstige Wertpapiere | 7 Verbindlichkeiten in Fremdwährung gegenüber Ansässigen im Euro-Währungsgebiet | |
| 8 Forderungen in Euro an öffentliche Haushalte | 8 Verbindlichkeiten in Fremdwährung gegenüber Ansässigen außerhalb des Euro-Währungsgebiets | |
| 9 Sonstige Aktiva | | |
| | 9 Ausgleichsposten für vom IWF zugeteilte Sonderziehungsrechte | |
| | 10 Sonstige Passiva | |
| | 11 Ausgleichsposten aus Neubewertung | |
| | 12 Kapital und Rücklagen | |
| Bilanzsumme | Bilanzsumme | |

| | |
|---|---|
| grün | Passivtausch zur geldpolitischen Neutralisierung ... |
| lila | ... von ESZB-Programm-Anleihekäufen (Fall a) |
| blau | ... von Devisenankäufen (Fall b) |
| grau | ... offener monetärer Staatsfinanzierung durch fiskalisch motivierte Staatsanleiheankäufe (Fall c) |

Quelle: EZB-Jahresbericht 2020, eigene Darstellung.

Die in der konsolidierten Bilanz des Eurosystems thematisch relevanten Aktiva-Positionen sind die programmgesteuerten Anleihekäufe des ESZB.[5] Diese werden offiziell mit einer Störung des geldpolitischen Transmissionsmechanismus begründet und erscheinen deshalb unter der Bilanzposition A7.1 „Zu geldpolitischen Zwecken gehaltene Wertpapiere". Gemäß internen Quellen der EZB wird jedoch auch die Rückführung von ansteigenden Zinsen für mediterrane Staatspapiere hervorgehoben, die ein zunehmendes Ausfallrisiko anzeigen würden (Randow und Neumann, 2021). Ankäufe mit dem Ziel einer Nivellierung der Risikoprämien oder gar einer so vermiedenen Staatsinsolvenz wären demnach als fiskalisch bzw. wirtschaftspolitisch zu werten, d. h. als nicht-geldpolitisch motivierter Eingriff. Insofern kämen auch andere Bilanzpositionen grundsätzlich infrage. SMP-, PSPP- und PEPP-Ankäufe könnten dann unter A7.2 „Sonstige Wertpapiere", ABSPP- und CBPP-Ankäufe unter A6 „Sonstige Forderungen in Euro an Kreditinstitute im Euro-Währungsgebiet" sowie unter A7.2, CSPP-Ankäufe ebenfalls unter A7.2 aufgeführt werden (Hansen und Meyer, 2019, 21). EZB-Bonds sind als Passiva-Position P4 „Verbindlichkeiten aus der Begebung von Schuldverschreibungen" bilanziell bereits vorbereitet. Mit ihrer Ausgabe würde die EZB Zentralbankgeld zurückholen, das im Rahmen der ESZB-Anleihekäufe neu geschaffen wurde [P1 „Banknotenumlauf"; den Hauptanteil ausmachend P2.1 „Einlagen auf Girokonten (einschließlich Mindestreserveguthaben)"; P2.2 „Einlagefazilität"].

### Bilanzverlängerung aufgrund von Devisenankäufen

Die Situation hier wäre ein Zustrom von ausländischem Kapital in den Geschäftsbankensektor infolge von Zinsarbitrage, Aufwertungserwartungen, eines Exportüberschusses oder Kapitalflucht. Die Notenbank könnte im Rahmen einer Wechselkursintervention Devisenankäufe vornehmen, um einer Aufwertung der eigenen Währung entgegenzuwirken. Dann wäre die Emission eigener Schuldverschreibungen eine Möglichkeit, die damit verbundene Liquiditätserhöhung abzuschöpfen.[6] Im Ergebnis käme es zu einer Bilanzverlängerung. EZB-Bonds würden als Passiva-Position P4 „Verbindlichkeiten aus der Begebung von Schuldverschreibungen" emittiert, um Liquidität in Form von Geschäftsbankeinlagen (P2.1 und P2.2) zu absorbieren, die zuvor als Gegenposition zu angekauften Devisen bzw. auf Devisen lautenden Forderungen (A2 und A3) entstanden sind. Gegebenenfalls bieten sich hier auch Notenbank-Schuldverschreibungen in Fremdwährung an (Rule, 2011, 10 f.; Schweizerische Nationalbank, 2011, 63).[7]

### Bilanzverlängerung aufgrund von fiskalisch motivierten Staatsanleiheankäufen

Die Höhe ihrer Staatsverschuldung könnte den weiteren Zugang zum Kapitalmarkt einzelner Euro-Mitgliedstaaten gefährden. Will man eine offene Staatsinsolvenz vermeiden und sollten die fiskalischen Rettungsschirme nicht ausreichen, könnte die EZB – entgegen ihrem Mandat (Art. 127 AEUV) – Staatsanleihen gefährdeter Staaten dauerhaft aufkaufen und „einfrieren". EZB-Bonds (P4) würden sie als de facto Euro-Bonds unter A7.2 „Sonstige Wertpapiere" geldpolitisch neutralisieren. Die EZB gerät zur direkten Finanziererin von Staaten.

## Spezielle Aspekte von Notenbank-Schuldverschreibungen in der EWU

Gemäß Art. 20 Abs. 1 ESZB-Satzung kann der „EZB-Rat ... mit der Mehrheit von zwei Dritteln der abgegebenen Stimmen über die Anwendung anderer Instrumente der Geldpolitik entscheiden, die er bei Beachtung des Artikels 2 für zweckmäßig hält". Dementsprechend verweist die Leitlinie (EU) 2015/510 der EZB über die Umsetzung des geldpolitischen Handlungsrahmens des Eurosystems (EZB/2014/60) an verschiedenen Stellen auf die Emission von EZB-Schuldverschreibungen.[8] Neben der Begründung (Nr. 9) wird in Art. 5 auf die Emission von EZB-Schuldverschreibungen als eines der möglichen Instrumente für Offenmarktgeschäfte verwiesen. In Art. 2 (Begriffsbestimmungen) heißt es dazu in Abs. 47: „‚Emission von EZB-Schuldverschreibungen' (issuance of ECB debt certificates) bezeichnet ein geldpolitisches Instrument, das für die Durchführung von Offenmarktgeschäften eingesetzt wird, wobei die EZB Schuldverschreibungen emittiert, die eine Schuldverpflichtung der EZB im Hinblick auf den Zertifikatsinhaber darstellen". Sie stellen somit eine Verbindlichkeit der EZB gegenüber den Inhaber:innen der Schuldverschreibungen dar (Art. 13 Abs. 1). Abgesehen von Altbeständen an Schuldverschreibungen der nationalen Zentralbanken zu Beginn der dritten Stufe der Euro-

5 Zu den ESZB-Anleihekaufprogrammen zählen: 1. Securities Markets Programme (SMP), 2. Covered Bond Purchase Programme 1-3 (CBPP 1-3), 3. Corporate Sector Purchase Programme (CSPP), 4. Asset-Backed Securities Purchase Programme (ABSPP), 5. Public Sector Purchase Programme (PSPP) und 6. Pandemic Emergency Purchase Programme (PEPP).

6 Allerdings könnte die Ausgabe von attraktiven EZB-Bonds grundsätzlich selber zu einem (weiteren) Devisenzustrom führen.

7 Abweichend von den Regularien der Schweizerischen Nationalbank scheinen EZB-Bonds nur in Eurodenomination möglich zu sein. Siehe Art. 39 Abs. 1 d, Leitlinie (EU) 2015/510 der EZB über die Umsetzung des geldpolitischen Handlungsrahmens des Eurosystems (EZB/2014/60).

8 In der konsolidierten Bilanz des Eurosystems gibt es bereits eine eigene Position P4 hierfür: „Verbindlichkeiten aus der Begebung von Schuldverschreibungen", die derzeit mit null ausgewiesen wird.

päischen Währungsunion (EWU), die das ESZB bis 2004 vollständig auflöste, hat die EZB von dem Instrument eigener EZB-Bonds bis dato keinen Gebrauch gemacht. Die nachfolgenden Ausführungen behandeln einzelne Problemaspekte von Notenbank-Schuldverschreibungen und fokussieren auf die besondere Situation der EWU.[9]

## Kapitalmarktunion und Reservewährung

Mit der Emission von EZB-Bonds wird die Kategorie „sichere Wertpapiere (mit kurzer Laufzeit)" um ein neues Angebot erweitert (Hardy, 2020, 10 ff.). Im Unterschied zu Staatsanleihen hoher Bonität, die auf segmentierte Märkte mit zum Teil geringer Liquidität treffen, bietet eine die gesamte Währungsunion einbeziehende Zinsstrukturkurve ein Referenzpreissystem, das auf ein breites Interesse der Kapitalanlegenden stoßen dürfte. Wenngleich die Beschränkung der EZB-Bonds auf Laufzeiten unter 12 Monaten die Zinsstrukturkurve auf kurze Durationen eingrenzt, wird eine angestrebte Euro-Kapitalmarktunion generell befördert. Gerade in unsicheren Zeiten können EZB-Bonds zu einer Marktstabilisierung beitragen, denn sie sind schwankenden Risikoprämien der Staaten weitgehend entzogen. Insbesondere für den Finanzsektor bieten sie einen erheblichen Sicherheitsgewinn. Denn bislang fand eine Verkoppelung von Liquiditätskrisen des eigenen Staates mit der Instabilität des Bankensektors durch eine enge finanzielle Verflechtung statt. Erhebliche Wertverluste der dort lagernden Staatspapiere infolge eines Anstiegs der Risikoprämien führten im Bankensektor zur Aufzehrung des Eigenkapitals. EZB-Bonds würden beide Sektoren entkoppeln. Als Repo-fähige Wertpapiere stießen sie auf eine breite, auch internationale Nachfrage. Sie beinhalten allenfalls Währungsrisiken für ausländische Anleger. Beispielsweise wären sie international eine konkurrierende Anlage zu kurzfristigen amerikanischen Staatsanleihen (Treasury Bills, T-Bills). Letztendlich könnte der Euro als internationale Transaktions- und Anlagewährung gewinnen (Boonstra, 2019, 10 f.).

## Quasi-sichere Wertpapiere: Konkurrenz zu Wertschriften der Staaten

Notenbank-Schuldverschreibungen sind ein quasi-ausfallsicheres Wertpapier, denn Notenbanken können nicht insolvent gehen (Hardy, 2020, 13 und 16). Indem sie ein gesetzliches Zahlungsmittel emittieren, sind sie jederzeit solvent und können auch bei negativem Eigenkapital ihre grundlegenden Funktionen erfüllen. Generell ist die Nachfrage nach sicheren Anleihen speziell seitens der Banken und Versicherungen sehr groß. Zudem ist das frei zugängliche Angebot an Staatsanleihen hoher Bonität durch die Anleiheankaufprogramme des ESZB relativ knapp, was deren Renditen teils ins Negative geführt hat. Dabei stehen EZB-Bonds im Wettbewerb insbesondere zu Staatsanleihen der bislang als ausfallsicher geltenden Mitgliedstaaten, also beispielsweise der von Deutschland, Österreich und den Niederlanden. Diese Staaten müssten zukünftig gegenüber EZB-Bonds eine gewisse Risikoprämie kalkulieren. Speziell für die potenziell ausfallgefährdeten Euroländer könnte diese Konkurrenz sogar einen spürbaren Anstieg der Spreads auslösen (Belke, 2010a, 18 f.; ders., 2010b, 360).[10] In Südkorea versucht man offenbar, diese Konkurrenz zu berücksichtigen, indem die Emission von Staats- und Zentralbank-Anleihen hinsichtlich Volumen, Auktionsdatum und Duration koordiniert wird. Das dürfte aber für die Eurozone kaum möglich sein, da es bei 19 Mitgliedstaaten zu viele Emittenten und Emissionen gibt. Indem EZB-Bonds eine maximale Laufzeit von einem Jahr haben, könnten Euro-Staaten die Konkurrenz durch eine Konzentration ihrer Emissionen auf die mittlere und lange Laufzeit reduzieren.[11] Schließlich ist ein zinsmindernder Aufwertungsdruck auf den Euro zu erwarten, denn auch für Nicht-Eurohalter:innen wären die EZB-Bonds attraktiv.[12]

Die herausgestellte Sicherheit von EZB-Bonds mag sich jedoch aus zwei Gründen als trügerisch erweisen. Denn insbesondere im Fall der EWU ist die Ausgabe von EZB-Bonds eher der Not geschuldet, dass alternativ infrage kommende Verkäufe von Staatsanleihen der Mitgliedstaaten kaum getätigt werden können, ohne unerwünschte Nebeneffekte hervorzurufen. Speziell die Schuldensituation einiger mediterraner Mitgliedstaaten scheint eine Art Sperrklinkeneffekt beim Ankauf dieser Anleihen in der EZB-Bilanz auszulösen. Von daher ist die autonome geldpolitische Steuerungsfähigkeit der EZB ohne den Einsatz von Notenbank-Schuldverschreibungen nur eingeschränkt bis kaum mehr vorhanden. Sie ist nicht mehr unabhängig in ihrem Handeln. Hinzu kommt, dass Euro-Staatsinsolvenzen zukünftig keinesfalls ausgeschlossen sind und damit verbunden eine gewisse Instabilität der Währungsunion immanent ist. Der Euro-Austritt eines Mitgliedstaats und die damit verbundene Herauslösung seiner nationalen Zentralbank aus dem Kreis der Eurostaaten macht die Einlösung der eingegangenen Ver-

9 Einen historischen Überblick bieten Hansen und Meyer (2021b).

10 Boonstra (2019, 11) sieht die Auswirkungen auf die Zinsspreads hingegen als kaum prognostizierbar an und plädiert daher für zunächst möglichst kurze Durationen. Hinzu kommt, dass die Notenbank den Zins geldpolitisch setzt, während die Staaten fiskalisch an der Aufrechterhaltung des Liquiditätszugangs und niedrigen Zinskosten interessiert sind (Rule, 2011, 18).

11 Gleichwohl würden sich die im Zeitablauf sinkenden Restlaufzeiten der Staatsanleihen irgendwann mit den Laufzeiten der EZB-Bonds mit niedrigeren Risikoprämien überschneiden.

12 Vgl. Boonstra (2019, 10 f.). Damit würde im Umfang des Zustroms von Auslandswährung eine Absorption entfallen.

pflichtungen zumindest unsicher. In dieser Situation würde eine Risikoprämie auf EZB-Bonds eine Indikatorfunktion übernehmen und im Krisenfall gegebenenfalls einen Circulus vitiosus auslösen können.

Im Ergebnis übernimmt die EZB mit der Emission eigener Schuldverschreibungen erhebliche Risiken in ihre Bilanz. EZB-Bonds werden zum Substitut für Eurobonds.[13] Es kommt zu einer Nationalisierung der Geldpolitik durch Staatsanleihekäufe, die die EZB neutralisiert und zugleich sozialisiert. Dies wird verstärkt, wenn die Ankäufe nicht dem EZB-Kapitalschlüssel folgen, sondern Krisenländer höher gewichten.[14] Die EZB würde zur Fiskalagentin für Länder mit erschwertem Liquiditätszugang. Einher gehen Nachteile auch für andere Eurostaaten, da die EZB-Bonds generell die Renditen steigen lassen.

### EZB-Bonds werden verzinst – Auswirkungen auf die Seigniorage

Im einfachen Fall eines Passivtausches wird ein Liquiditätsüberschuss durch die Ausgabe einer verzinsten Notenbank-Schuldverschreibung gegen Überschussreserven des Bankensektors abgebaut. Da die Überschussreserven im Regelfall unverzinst oder niedriger verzinst sind als die Notenbank-Schuldverschreibung mit einer vergleichsweise längeren Laufzeit, mindert diese Strategie den Notenbankgewinn.[15] Im Fall einer Liquiditätsabschöpfung durch EZB-Bonds als Alternative zum Verkauf von Anleihen des PSPP- bzw. PEPP-Programms kommt es zu einem positiven Seigniorage-Differenzgewinn. Staatsanleihen mit teils erheblichen Risikoprämien bleiben in der Bilanz, stattdessen werden niedriger verzinste Notenbank-Schuldverschreibungen ausgegeben (Tonveronachi, 2014, 8 f.; ders., 2015, 4 ff.)· Allerdings dürften die Risikoprämien die langfristigen Risiken eines Zahlungsausfalls eher zu gering ausweisen, da die Risikobewertung des Marktes die fiskalischen und monetären Rettungsschirme bereits einpreist und somit einen verzerrten Indikator darstellt.

Sodann blieb die EZB-Gewinnverteilung in den obigen Aussagen bislang außen vor. Die monetären Einkünfte der nationalen Zentralbanken und der Nettogewinn bzw. -verlust der EZB wird entsprechend dem EZB-Kapitalschlüssel an die nationalen Zentralbanken verteilt (Art. 32.5 und 33 ESZB-Satzung). Im Regelfall wird der Überschuss der nationalen Zentralbanken, gegebenenfalls korrigiert um Wagnisrückstellungen und Rücklagenzuführungen, an den Haushalt des Nationalstaates verteilt. Von daher stellt die Staatsanleiheverzinsung prinzipiell einen durchlaufenden Posten in den Bilanzen der Notenbanken dar. Insofern besteht ein fiskalisches Interesse am Einsatz von EZB-Bonds, die einen quasi zinslosen Staatskredit ermöglichen.

### Dauerhafte Einlagerung von Staatsschulden – EZB-Bonds als Euro-Bonds

Die Staatsanleihekäufe im Rahmen der „außergewöhnlichen Geldpolitik“ würden zwecks Rückholung der Liquidität zu gegebener Zeit ihren Verkauf bedingen bzw. keine Wiederanlage von Tilgungsbeträgen fällig werdender Anleihen zur Konsequenz haben. Um den Tatbestand einer monetären Staatsfinanzierung auszuschließen, hat das Bundesverfassungsgericht in seinem Urteil vom 5. Mai 2020 (BVerfG, Urteil 2 BvR 859/15) dementsprechend sieben Prüfkriterien formuliert. Unter anderem müssen die „Ankäufe begrenzt oder eingestellt und erworbene Schuldtitel wieder dem Markt zugeführt werden ... , wenn eine Fortsetzung der Intervention zur Erreichung des Inflationsziels nicht mehr erforderlich ist“.[16] In der Anwendungspraxis der PSPP- und PEPP-Programme ist der Regelfall allerdings eine Haltung bis zur Endfälligkeit mit Wiederanlage der Tilgungsbeträge. Indem das Eurosystem die Wertpapiere endgültig kauft und die Kredite faktisch prolongiert, werden Staatsschulden dauerhaft in den Bilanzen des ESZB belassen. Der Einsatz von EZB-Bonds, der den Bestand dieser Anleihen im Fall einer restriktiven Geldpolitik unverändert lässt, wäre die logische Konsequenz eines solchen Bestrebens.[17]

Indem dieses Handeln der EZB mit Rücksicht auf drohende Kursverluste und Instabilitäten des Banken- und Versicherungssektors geldpolitisch als Second-Best-Lösung gerechtfertigt werden kann, ließen sich Notenbank-Schuldverschreibungen auch ganz offen fiskalpolitisch einsetzen. So könnte eine Notenbank eine kürzlich emittierte Staatsanleihe ohne Endfälligkeit am Kapitalmarkt erwerben und die durch den Kaufpreis abgeflossene Liquidität durch einen Passivtausch mit einem NB-Bonds neutralisieren. Damit wäre die Staatsschuld mit unendlicher Laufzeit bei der Notenbank „auf ewig eingefroren“ –

13 Ähnlich argumentiert Boonstra (2019, 11).
14 Vgl. hierzu die Untersuchung von Hansen und Meyer (2020b). Siehe auch Belke (2010b, 360 f.).
15 Hieran ändern auch negative Verzinsungen nichts. Um die Belastungen gering zu halten, würde die Notenbank eigene Schuldverschreibungen mit geringer Duration herausgeben. Vgl. Belke (2010a, 21). Dalton und Dziobek (2005, 8 ff.) berichten, dass einige Zentralbanken, wie die von Brasilien, Chile und Korea erhebliche Verluste erfahren haben.
16 BVerfG, Urteil 2 BvR 859/15 v. 5. Mai 2020, Rn. 216.
17 Sollte es hingegen zur Tilgung der Staatsanleihen zum Zeitpunkt ihrer Fälligkeit ohne Wiederanlage kommen, fließt der EZB Liquidität zu, was gegebenenfalls durch den Rückkauf von EZB-Bonds neutralisiert werden könnte.

de facto eine monetäre Staatsfinanzierung, denn sie wurde in eine Verbindlichkeit der Notenbank umgewandelt.[18]

In einer Währungsunion ist die zentrale Notenbank nicht die eines Souveräns. Im Fall der EWU ist die EZB die Notenbank aller 19 Euromitgliedstaaten.[19] Hieraus ergeben sich unter Umständen Verteilungs- und Free-Rider-Probleme. So ist im Fall der PSPP- bzw. PEPP-Ankäufe eine spezielle Portfolioallokation und Haftung vorgesehen. Mit Ausnahme der Schuldtitel internationaler Organisationen (10 %-Anteil) und den EZB-Ankäufen von vorwiegend nationalen Staatstiteln (10 %-Anteil), für die bei etwaigen Verlusten gemeinschaftlich gehaftet wird, haften die nationalen Zentralbanken für ihre Staatsanleihekäufe (80 %-Anteil) jeweils selbst.[20] Folgerichtig wäre es daher, EZB-Bonds nur bis zur Höhe des 10 %- bzw. gegebenenfalls 20 %-Anteils zu emittieren – dem Anteil, für den eine Risiko- und Haftungsvergemeinschaftung besteht (Boonstra, 2019, 11). Ansonsten würden EZB-Bonds – vergemeinschaftlichte Verbindlichkeiten der Euro-Staaten – ausgegeben, um die national angekaufte und endgültig eingelagerte Staatsschuld zu finanzieren. Staatsanleihen der Nationalstaaten würden zu EZB-Bonds im Sinne von Eurobonds mit gemeinschaftlicher Haftung. Diese Risikovergemeinschaftung würde insbesondere im Fall einer Staateninsolvenz und/oder eines Austritts aus der Währungsunion aktuell. Alternativ könnten die nationalen Zentralbanken im Umfang ihrer Haftung bzw. Ankäufe eigene Bonds emittieren. Dies ist derzeit aus gutem Grund jedoch nicht vorgesehen [Art. 13 Leitlinie (EU) 2015/510 der EZB über die Umsetzung des geldpolitischen Handlungsrahmens des Eurosystems (EZB/2014/60)], denn damit würde die gemeinsame Geldpolitik zugunsten nationalfiskalischer Zielsetzungen nationalisiert (Hardy, 2020, 17).

Weitergehende Vorschläge gründen auf der Absicht, das Maastrichter Konstrukt einer Währungsunion ohne Fiskalunion mit EZB-Bonds zu heilen.[21] Hierzu müsste die EZB nationale Staatsanleihen entsprechend dem EZB-Kapitalanteil von Banken aufkaufen – bei gleichzeitiger Emission von gemeinschaftlichen EZB-Bonds. Die finanziellen Intermediäre erhalten ein risikoloses, homogenes Wertpapier, die Offenmarktpolitik würde vereinfacht und die Kapitalmarktunion wäre vollendet. Zudem würde der Teufelskreis aus Staatsschuldenkrise und Bankenkrise durchbrochen, da die EZB-Bonds kein Emittentenrisiko tragen. Erkauft wird der Stabilitätsgewinn allerdings durch eine Risikovergemeinschaftung (Euro-Bonds), denn der Zahlungsausfall eines Landes trifft die EZB und geht zulasten des an die nationalen Zentralbanken zu verteilenden EZB-Gewinns. Zwecks Einhaltung der 60 %-Fiskalregel wird zudem vorgeschlagen, dass die EZB nur die von ihr ausgegebenen Bonds als Sicherheit gegenüber Banken akzeptiert (Tonveronachi, 2015, 4 ff.). Durch Einführung einer entsprechend geänderten Fiskalregel, die die Emission von EZB-Bonds mit dem Schuldenstand eines Landes verknüpft, würden die Staaten bei Überschreitung sanktioniert, indem für Banken ein Erwerb von Staatstiteln unattraktiver würde. Damit würde die EZB jedoch eine nicht zu ihren Aufgaben zählende Kontroll- bzw. Steuerungsfunktion der Schuldenaufnahme von Staaten übernehmen.

## Fazit

Notenbank-Schuldverschreibungen sind in der Situation eines Liquiditätsüberschusses ein mögliches Instrument zur Liquiditätsabschöpfung. Auch die EZB sieht in ihrer Leitlinie (EU) 2015/510 über die Umsetzung des geldpolitischen Handlungsrahmens des Eurosystems (EZB/2014/60) die Emission von EZB-Bonds vor. Angesichts einer etwaigen, inflationsbedingten Notwendigkeit der Rückführung der Überschussliquidität des Bankensektors und den Gefahren einer aus massiven Verkäufen von Staatsanleihen der PSPP- und PEPP-Bestände erwachsenden Instabilität, könnte die EZB die Möglichkeit zur Emission eigener Schuldverschreibungen ergreifen. Als quasi-ausfallsichere Wertschriften würden sie auf eine hohe Nachfrage stoßen, eine unionsweite Zinsstrukturkurve entwickeln helfen und zur Kapitalmarktunion beitragen. Ihre Konkurrenz zu Staatsanleihen könnte generell einen Anstieg der Risikoprämien bewirken und insbesondere für Hochschuldenländer die Zinslasten steigen lassen. Zudem besteht die Gefahr, dass es zu einer dauerhaften Einlagerung von Staatsschulden aus fiskalischen Motiven kommt. Infolge der Gewinn- bzw. Verlustverteilung im ESZB-System würden EZB-Bonds zu Euro-Bonds mit gemeinschaftlicher Haftung. Weitere Umverteilungseffekte könnten im Zusammenhang mit den PSPP- und PEPP-Ankäufen entstehen, insbesondere sofern die Staatsanleihekäufe der nationalen Zentralbanken durch EZB-Bonds neutralisiert werden.

18 Entsprechende Ansätze finden sich im PSPP- und PEPP-Programm. So sind Restlaufzeiten der angekauften Wertpapiere des PSPP-Programms von bis zu 30 Jahren für eine geldpolitische Maßnahme eher unüblich (Art. 3 Abs. 3 Beschluss (EU) 2020/188 der EZB). Zudem ist eine Wiederanlage von Tilgungsbeiträgen vorgesehen (Art. 6 Abs. 3 Beschluss (EU) 2020/188 der EZB).

19 Konkret setzt sich das ESZB aus den Notenbanken aller 27 EU-Mitgliedstaaten zusammen, wobei die 19 Euro-Mitgliedstaaten das Eurosystem bilden und besondere Rechte und Pflichten haben (Art. 282 AEUV). Vgl. auch Boonstra (2019, 11).

20 Siehe Art. 6 Abs. 1 und 2 Beschluss (EU) 2020/188 der EZB und Art. 1 Abs. 2 Beschluss (EU) 2020/440 der EZB. „Im Ergebnis unterliegen somit weiterhin 20 % der Ankäufe von Vermögenswerten im PSPP dem Prinzip der Risikoteilung, während die Risikoteilung für 80 % der erworbenen Titel ausgeschlossen ist." Deutsche Bundesbank (o. J.).

21 So Tonveronachi (2014, 4 und 6); zur Umsetzung vgl. 8. Während Tonveronachi keine Notwendigkeit zur Änderung der EU-Verträge sieht, dürfte sein Vorschlag eine nicht vertragskonforme monetäre Staatsfinanzierung darstellen.

**Title:** ***ECB Debt Certificates – New Use for an Old Instrument?***

**JEL Classification:** *E44, E52, E58, E63, H63*
